# Primary peritoneal ectopic pregnancy: A case report

**DOI:** 10.1016/j.ijscr.2022.107847

**Published:** 2022-12-23

**Authors:** Hassan M. Elbiss, Abeer Al Tahrawi, Fikri M. Abu-Zidan

**Affiliations:** a Department of Obstetrics and Gyanecology, College of Medicine and Health Sciences United Arab Emirates University, Al-Ain, United Arab Emirates; b Department of Pathology, College of Medicine and Health Sciences United Arab Emirates University, Al-Ain, United Arab Emirates; c Department of The Research Office, College of Medicine and Health Sciences United Arab Emirates University, Al-Ain, United Arab Emirates

**Keywords:** Ectopic pregnancy, Primary peritoneal pregnancy, Laparoscopy

## Abstract

**Introduction:**

Primary peritoneal ectopic pregnancy is a rare condition that can be life-threatening. Herein, we report such a case which was managed by laparoscopy.

**Presentation of the case:**

A 31-year-old G1P0 woman, who had a history of pelvic infection and primary infertility, presented with lower abdominal pain and mild vaginal spotting. Abdominal and bimanual pelvic examination revealed mild left pelvic tenderness. Her serum β-human chorionic gonadotropin (β-HCG) was 7247 IU. Transvaginal ultrasound demonstrated a mass measuring around 1.5 cm in diameter with a well-defined yolk sac adherent to the left ovary. A left fallopian tube ectopic pregnancy was suspected. Laparoscopy revealed that both fallopian tubes were normal and freely moving. Peritoneal ectopic pregnancy was seen behind the uterus which was removed laparoscopically. Histopathology confirmed the diagnosis. The patient had a smooth postoperative recovery.

**Discussion:**

Primary peritoneal pregnancy can be life-threatening. A thorough laparoscopic examination of the entire pelvis and abdomen should be done by an experienced surgeon when the location of the suspected ectopic pregnancy could not be identified.

**Conclusion:**

Diagnostic laparoscopy for ectopic pregnancy should include the whole pelvis and the accessible part of the abdomen when the tubes and ovaries are normal.

## Highlights

• Untreated primary peritoneal ectopic pregnancy can cause life-threatening bleeding.
• Death from ectopic pregnancy is due to delayed presentation or missed diagnosis.
• Primary peritoneal pregnancy carries more maternal morbidity and mortality.
• High index of suspicion is needed for timely diagnosis of ectopic pregnancy.

## 1 Introduction

Ectopic pregnancy is defined as the implantation of pregnancy outside the endometrial cavity. It is common during the first trimester and is one of the leading causes of maternal mortality which ranges from 2 % to 2.5 % [1], [2]. Its commonest site is the fallopian tubes (95 %) [3].

Primary peritoneal ectopic pregnancy, in which the gestational sac attaches directly to the abdominal peritoneum, is rare (9 in every 1000 ectopic pregnancies) [4]. In most cases, the mother and fetus have a poor prognosis. If the gestational sac ruptures, it may cause severe bleeding having a high mortality reaching up to 20 % [5], [6], [7]. Sensitive pregnancy tests can detect low levels of β-human chorionic gonadotropin (β-HCG). Around 7–10 % of all pregnancies are classified as pregnancies of unknown location (PUL) [8], [9], [10]. Most of these pass unnoticed without complications. Some of them may have small foci of cells secreting β-HCG which may grow to macroscopic ectopic pregnancy and cause life-threatening bleeding. Herein, we present a case of primary peritoneal pregnancy which was successfully diagnosed and managed. This work has been reported in line with the SCARE criteria [11].

## 2 Presentation of case

**Fig. 1:**
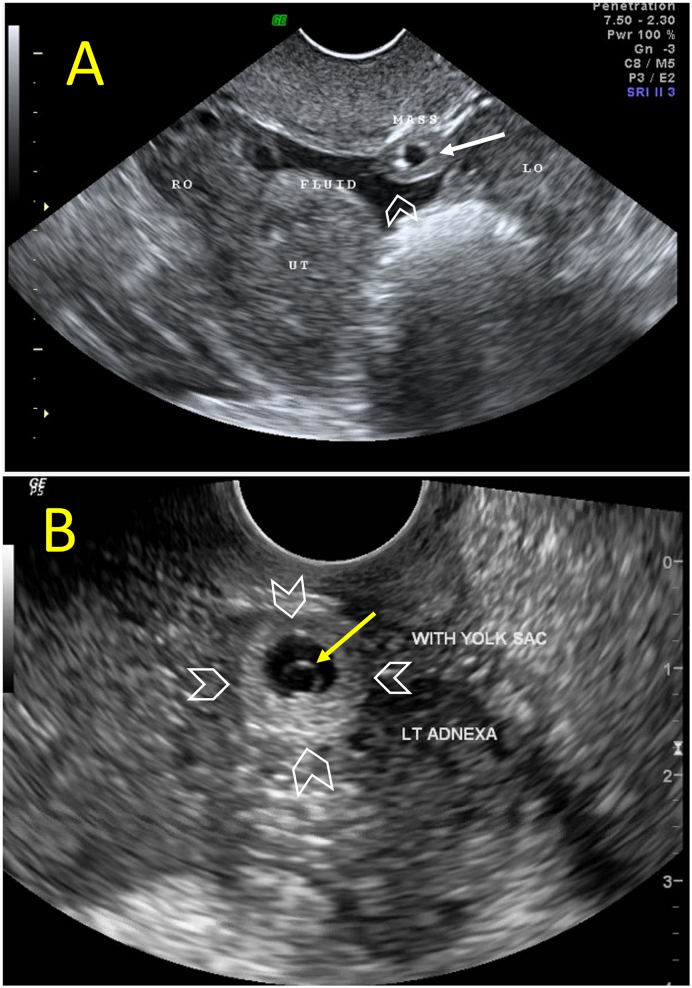
Transvaginal ultrasound scan. Transvaginal ultrasound scan revealed absence of intrauterine gestational sac. A complex mass measuring 1.2 × 0.9 × 1 cm was seen (white arrow) close to the left ovary (LO) and was suspected to be a gestational sac at the left adnexa There was minimal fluid in the pouch of Douglas (arrow head) (A). Follow up transvaginal ultrasound 4 days later showed that the mass has increased in size (1.49 × 1.19 × 1.44 cm) (arrow heads) which contained a well-defined yolk sac (yellow arrow) (B). (For interpretation of the references to colour in this figure legend, the reader is referred to the web version of this article.)

**Fig. 2:**
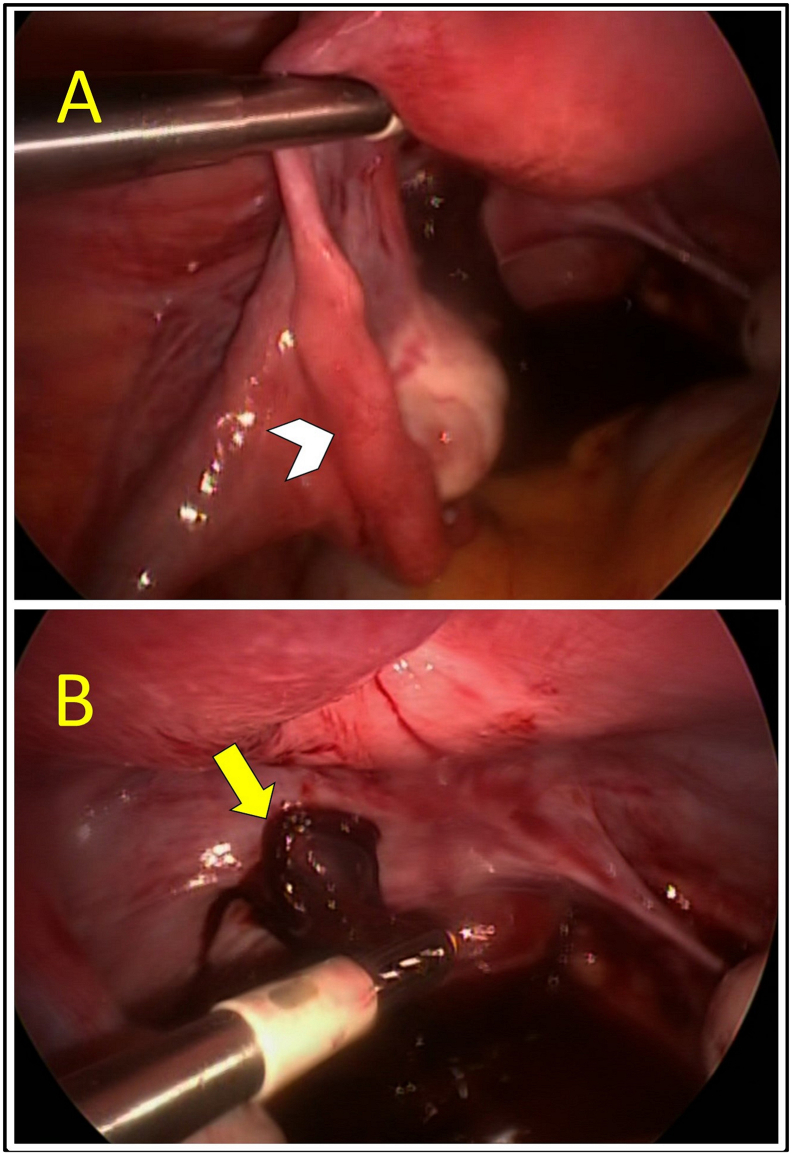
Laparoscopic findings. Laparoscopy showed that the left fallopian tube (arrow head) and ovary were normal and mobile (A) both ovaries were normal and mobile. Ectopic pregnancy of 2 cm wide having minimal bleeding was located on the left posterior wall of the peritoneum behind the uterus 1 cm above the uterosacral ligament (arrow head) (B).

**Fig. 3:**
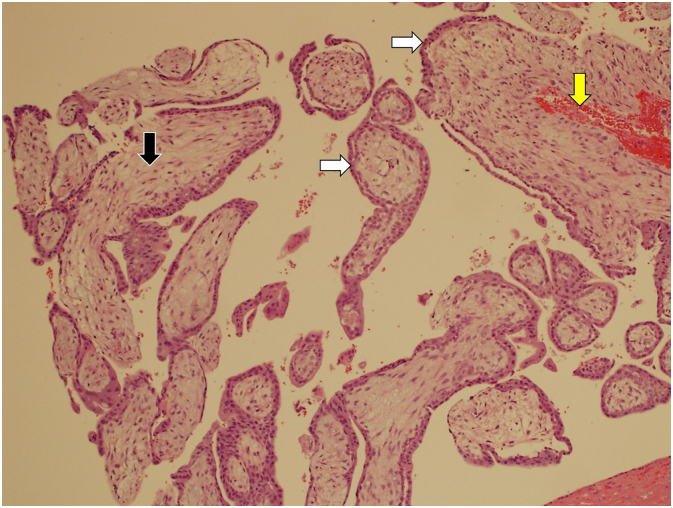
H & E stain. H & E stain, ×10 showing chorionic villi. Each villous is formed of a central mesenchymal core (black arrow) which is rimmed by syncytiotrophoblasts and cytotrophoblasts (white arrows). A central blood vessel is visualized (yellow arrow). (For interpretation of the references to colour in this figure legend, the reader is referred to the web version of this article.)

A 31-year-old G1P0 woman presented with lower abdominal pain and mild vaginal spotting. Her last menstrual period was 6 weeks before the presentation. Her period was regular and she had a history of pelvic infection and primary infertility. On admission, her blood pressure was 110/70 mmHg, her pulse was 96 beats per minute, and her temperature was 37.2 °C. Clinical examination revealed tenderness on the left lower abdomen, the uterine cervix was closed, and no bleeding was noted. A bimanual examination revealed mild pelvic tenderness on the left side. Her serum β-HCG was 7247 IU. Transvaginal ultrasound scan revealed a retroverted uterus, an endometrium thickness measuring 0.86 cm, with no intrauterine gestational sac. A complex mass measuring 1.2 × 0.9 × 1 cm was seen near the left ovary which was suspected to be a gestational sac of the left adnexa. There was minimal fluid in the pouch of Douglas (Fig. 1A). Follow up transvaginal ultrasound 4 days later showed that the mass has increased in size (1.49 × 1.19 × 1.44 cm) and contained a well-defined yolk sac. The intraperitoneal fluid did not increase in volume (Fig. 1B). The patient was diagnosed to have a left fallopian tube ectopic pregnancy. There was no relevant drug, family and psychosocial history. Accordingly, a decision was made to perform a therapeutic laparoscopy (undertaken by HM Elbiss). At laparoscopy the uterus was of normal size, both tubes looked normal with no evidence of tubal pregnancy (Fig. 2A), both ovaries were normal and mobile. Ectopic pregnancy of 2 cm wide having minimal bleeding was located on the left posterior wall of the peritoneum behind the uterus, 1 cm above the uterosacral ligament (Fig. 2B). As the β-HCG level was high (7247 IU), it was not advisable to consider methotrexate therapy because the risk of rupture of the ectopic and failure of the treatment would be high. The location of the ectopic pregnancy was behind the uterus between the right and left utero-sacral ligaments. Both ureters were identified. The ectopic pregnancy was not near either of them. The top of the peritoneal pregnancy was incised and evacuated, the base was cauterized, then excised with scissors, and sent for pathological examination. Histopathology confirmed the presence of large first trimester chorionic villi formed of stroma and covered by two layers of cells, cytotrophoblast and syncytiotrophoblast (Fig. 3). There was no major nerves passing near the ectopic pregnancy. Although the excision was superficial, it was adequate to completely remove the ectopic pregnancy. The patient had a good postoperative recovery and was discharged the next day. One week later, she was doing well and her β-HCG was 162 IU.

## 3 Discussion

The criteria for diagnosis of primary peritoneal ectopic pregnancy according to the modified Studdiford classification are: 1) the presence of a pregnancy less than 12 weeks in which trophoblastic attachments are related solely to the peritoneal cavity, 2) grossly normal tubes and ovaries, and 3) the absence of uteroperitoneal fistula [12]. All these criteria were present in our patient. Approximately 1–2 % of pregnancies are ectopic [13]. However, these pregnancies account for 3 % to 4 % of pregnancy-related deaths [14]. This mortality rate steadily declined during the last three decades [15] which was attributed to improvements in pregnancy testing, ultrasound diagnosis, and surgical management. Death from ectopic pregnancy is related to the delayed presentation or missed diagnosis. Primary peritoneal pregnancy carries more maternal morbidity and mortality compared with tubal pregnancy due to delayed diagnosis. During a diagnostic laparoscopy, a thorough inspection of the whole pelvis and abdomen is needed to exclude this condition. This diagnosis can be easily missed by evaluating only the fallopian tubes.

Similar cases have been reported before highlighting the same lessons we have learned. Yasmin et al. [16] reported a 29-year-old woman presenting with generalized abdominal pain and high B-HCG levels. Ultrasound excluded an intra-uterine gestation. Laparoscopy revealed hemoperitoneum and normal fallopian tubes and ovaries. An abnormal tissue in the left uterosacral ligament was removed which was confirmed by histopathology to be a ruptured ectopic pregnancy. Giovanni Di Lorenzo et al. [17] focused on the laparoscopic management of parametrial ectopic pregnancy being a safe procedure. The recommendations of these two case reports are very similar to ours. Our case demonstrates the importance of looking for ectopic pregnancy outside the tubes. During laparoscopy, careful assessment of the pelvis and accessible part of the abdomen is needed when both tubes and ovaries are normal. Obviously, this needs a high level of skills, therefore senior and skilful backup is needed for less experienced surgeons who are performing diagnostic laparoscopy for ectopic pregnancy and could not find the source of bleeding.

## 4 Conclusion

Primary peritoneal pregnancy is an extremely rare condition. High index of suspicion is needed for timely diagnosis. Diagnostic laparoscopy for ectopic pregnancy should include the whole pelvis and the accessible part of the abdomen when the tubes and ovaries are normal.

## Informed consent

The patient gave her informed written consent to publish her case and clinical images.

## Ethical approval

N/A.

## Funding

N/A.

## Author contribution

Hassan M Elbiss: Preparation of manuscript.

Abeer Al Tahrawi: Assisted in histology part of the paper.

Fikri M. Abu-Zidan: Revised and modified manuscript.

## Guarantor

Hassan M Elbiss.

## Research registration number

N/A.

## Declaration of competing interest

N/A.
